# Enhancing Caffeic Acid Production in *Escherichia coli* Through Heterologous Enzyme Combinations and Semi-Rational Design

**DOI:** 10.3390/metabo16010062

**Published:** 2026-01-09

**Authors:** Qing Luo, Weihao Wang, Qingjing Huang, Chuan Wang, Lixiu Yan, Jun Kang, Jiamin Zhang, Jie Cheng

**Affiliations:** 1 Meat Processing Key Laboratory of Sichuan Province, College of Food and Biological Engineering, Chengdu University, Chengdu 610106, China; p2214931@mpu.mo (Q.L.); ventureh994@163.com (W.W.); zhangjiamin@cdu.edu.cn (J.Z.); 2 Chongqing Academy of Metrology and Quality Inspection, Chongqing 401123, China; 15803031339@163.com (Q.H.); cqjcylx@163.com (L.Y.); 3 Faculty of Applied Sciences, Macao Polytechnic University, Macao 999078, China; 4 The Key Laboratory of Natural Products and Functional Food Development of Luzhou, Sichuan Vocational College of Chemical Technology, Luzhou 646005, China; 13398286755@163.com

**Keywords:** caffeic acid, HpaB, substrate flexibility, semi-rational design

## Abstract

Background/Objectives: Caffeic acid is a hydroxycinnamic acid that has a wide range of applications in the medical field. The synthesis of caffeic acid using microbial fermentation technology is an environmentally friendly method. Methods: By engaging various enzymes, specifically 4-hydroxyphenylacetate 3-monooxygenase (HpaB), sourced from diverse bacterial strains, we successfully engineered a functional version of this enzyme within *Escherichia coli*, enabling the production of caffeic acid. In addition to the two common tyrosine ammonia lyases (TAL) and HpaC, different combinations of HpaB demonstrated varying abilities in converting the substrate L-tyrosine into the desired product, caffeic acid. Results: Under shake-flask culture conditions, the highest yield of caffeic acid was achieved with an enzyme mixture containing HpaB from *Escherichia coli*, reaching 75.88 mg/L. Enhancing the activity of the rate-limiting enzyme through engineering could potentially increase caffeic acid titer. This study aims to conduct a semi-rational design of HpaB through structure-based approaches to screen for mutants that can enhance the production of caffeic acid. Initially, the predicted three-dimensional structure of HpaB was generated using AlphaFold2, and subsequent analysis was conducted to pinpoint the critical mutation sites within the substrate-binding pocket. Five key amino acid residues (R113, Y117, H155, S210 and Y461) located in the vicinity of the flavin adenine dinucleotide binding domain in HpaB from *Escherichia coli* could be instrumental in modulating enzyme activity. Subsequently, the mutant S210G/Y117A was obtained by iterative saturation mutagenesis, which increased the titer of caffeic acid by 1.68-fold. The caffeic acid titer was further improved to 2335.48 mg/L in a 5 L fermenter. The findings show that the yield of caffeic acid was significantly enhanced through the integration of semi-rational design and fermentation process optimization.

## 1. Introduction

Advancements in bioinformatics, artificial intelligence, and synthetic biology have facilitated a deeper comprehension of enzymatic processes and the development of efficient industrial biotransformation platforms. Recently, numerous significant high-value chemicals have been successfully synthesized in microbial systems via the application of synthetic biology and enzyme engineering techniques, such as carminic acid [1], aspartate [2], ferulic acid [3], quercetin [4], guanidinoacetate [5], hydroxytyrosol [6], silybin [7], and catechol [8].

Caffeic acid, a naturally occurring polyphenolic compound, exhibits diverse biological activities and is widely applied in the fields of medicine and cosmetics. Its remarkable antioxidant properties help neutralize free radicals and inhibit lipid peroxidation, thereby delaying cellular aging and protecting tissues from oxidative stress [9]. Additionally, caffeic acid possesses anti-inflammatory capabilities, demonstrating therapeutic potential for various inflammatory diseases by modulating inflammatory signaling pathways [10]. It is also a focal compound in antimicrobial and anticancer studies, showing significant effects in inducing apoptosis in cancer cells and mitigating multidrug resistance [11]. Caffeic acid plays a crucial role in pharmaceutical synthesis as a precursor to other phenolic acids, such as chlorogenic acid [12] and rosmarinic acid [13]. Moreover, its ester derivatives, such as caffeic acid phenethyl ester, are noted for their enhanced bioactivity and are widely utilized in the development of novel therapeutic agents [14]. Existing production methods generally extract and purify caffeic acid from plants; however, the concentration of caffeic acid found within these plants is usually quite low (0.131%), and the process of extracting and purifying caffeic acid from plants is complex [15], resulting in low efficiency. With the increasing demand for caffeic acid in recent years, it is necessary to develop new methods to improve the production and conversion rate of caffeic acid. The production of caffeic acid using biomass as substrate and microbial fermentation method has attracted a lot of attention due to its environmentally friendly and simple process advantages.

The biosynthesis of caffeic acid is mainly achieved through two modules. First, carbon sources such as glucose produce intermediates like phosphoenolpyruvate (PEP) and 4-phosphoerythroase (E4P) through glycolysis and the pentose phosphate pathway, which, in turn, produce aromatic amino acids such as L-tyrosine and L-phenylalanine. In the second pathway, aromatic amino acids are deaminated to *p*-coumaric acid by phenylalanine ammonialase (PAL) or tyrosine ammonialase (TAL). The resultant *p*-coumaric acid is subsequently hydroxylated by the enzyme *p*-coumaric acid 3-hydroxylase (C3H), transforming it into caffeic acid. Liu et al. reported that TAL and different species of 4-hydroxyphenylacetate 3-monooxygenase (HpaB) and NADPH-flavin oxidoreductase (HpaC) were used to construct the pathway for caffeic acid synthesis in *S. cerevisiae* [16]. The different combinations of HpaB and HpaC presented varied capabilities in producing the target product, caffeic acid, from the substrate, L-tyrosine [16]. Rodrigues et al. achieved the first caffeic acid titer of 280 mg/L in *E. coli* using CYP199A2 and tyrosine as precursors [17]. Li et al. characterized the functional expression of plant-derived coumarate 3-hydroxylase and successfully achieved the de novo biosynthesis of caffeic acid within *S. cerevisiae* [18]. *E. coli* was able to achieve higher caffeic acid yields compared to *S. cerevisiae*, leading to its selection as the production system for the purposes of this study [19].

Several metabolic engineering approaches have been investigated to boost the synthesis of caffeic acid in *E. coli*, focusing on the optimization of precursor availability, enzyme function, and the secretion of the final product. Wang et al. obtained a caffeic acid yield of 7.92 g/L in a 5 L fermenter, which marked the reported yield among microorganisms using a simple carbon source, by enhancing the synthesis of the cofactor FAD and discovering new transporter proteins using transcriptome analysis [20]. Zhang et al. produced caffeic acid from glucose and xylose up to 106 mg/L by modulating the production medium and the copy number of biosynthetic genes [21]. Haslinger et al. evaluated the effect of different tyrosine ammoniacase and PratherP450/redox partner combinations on caffeic acid synthesis, and the final yield was increased by 10% [22]. Sakae et al. optimized the expression of key enzymes with precursor supply and produced 6.17 g/L caffeic acid at 90 h of culture [23]. By combining these strategies—precursor enhancement, enzyme optimization, cofactor supply improvement, and product secretion—significant increases in caffeic acid titers have been achieved in engineered *E. coli*, paving the way for more efficient biosynthetic production systems.

**Figure 1:**
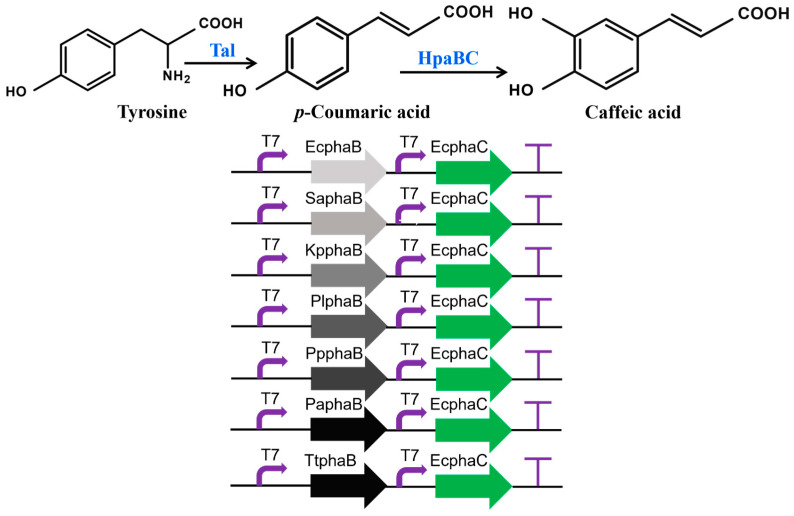
The heterogeneous pathway for caffeic acid production from tyrosine in *E. coli*.

In this study, a highly efficient synthetic route for the generation of caffeic acid was accomplished in *E. coli*, as depicted in Figure 1. Firstly, L-tyrosine was deaminated by Tal to generate *p*-coumaric acid. Subsequently, *p*-coumaric acid was hydroxylated by HpaBC to form caffeic acid. Next, the titer of caffeic acid was increased by inhibiting competing metabolic pathways through gene knockout. In this study, a semi-rational design was employed to isolate mutants exhibiting enhanced catalytic efficiency. Subsequently, the structure–function relationships of the chosen HpaBC mutants were investigated through molecular dynamics (MD) simulations, laying the groundwork for a theoretical understanding and offering initial insights crucial for the further engineering of HpaBC to satisfy industrial standards.

## 2. Materials and Methods

### 2.1. Strains, Plasmids, Medium and Chemicals

The bacterial strains and plasmids constructed in this study are presented in Table 1. *E. coli* DH5α was utilized for plasmid construction and maintenance, while *E. coli* BL21(DE3) served as the host for caffeic acid production. Luria–Bertani (LB) medium was used for strain activation and the production of caffeic acid. Zhou et al. used a strategy of error-prone PCR to screen for tyrosine ammonia lyase (TAL) mutants (RgTal) with stronger catalytic efficiency [24]. We synthesized this mutant RgTal through Sangon Biotech (Shanghai) Co., Ltd. The nucleotide sequences of HpaB from *E. coli*, NADPH-flavin oxidoreductase gene *hpaC* from *E. coli*, HpaB from *Sulfobacillus acidophilus*, HpaB from *Klebsiella pneumoniae*, HpaB from *Photorhabdus luminescens*, HpaB from *Pseudomonas putida*, HpaB from *Pseudomonas aeruginosa*, and HpaB from *Thermus thermophilus* are registered in GenBank under the accession numbers AAR11357.1, AAR11356.1, AEJ40622.1, CDO16163.1, AAO17197.1, ADA63516.1, PKG21040.1, and BFH87998, respectively. The codon-optimized hpaB gene from *Sulfobacillus acidophilus* (SahpaB, GenBank Acc. No. AEJ40622.1), the hpaB gene from *Klebsiella pneumoniae* (KphpaB, GenBank Acc. No. CDO16163.1), the hpaB gene from *Photorhabdus luminescens* (PlhpaB, GenBank Acc. No. AAO17197.1), the hpaB gene from *Pseudomonas putida* (PphpaB, GenBank Acc. No. ADA63516.1), the hpaB gene from *Pseudomonas aeruginosa* (PahpaB, GenBank Acc. No. PKG21040.1), and the hpaB gene from *Thermus thermophilus* (TthpaB, GenBank Acc. No. BFH87998) were examined. and RgTal were chemically synthesized and inserted into pTrc99a to form plasmid pTrc99a-RgTal-EcphaB-EcphaC, pTrc99a-RgTal-SaphaB-EcphaC, pTrc99a-RgTal-KpphaB-EcphaC, pTrc99a-RgTal-PlphaB-EcphaC, pTrc99a-RgTal-PpphaB-EcphaC, pTrc99a-RgTal-PaphaB-EcphaC and pTrc99a-RgTal-TtphaB-EcphaC. The integrity of all vector constructs was confirmed via Sanger sequencing performed by Sangon Biotech Co., Ltd., Shanghai, China, while the standard reagents were procured from Aladdin, Shanghai, China.

### 2.2. Culture Medium and Conditions

Shake-flask fermentation experiments were conducted in 250 mL Erlenmeyer flasks, each containing 25 mL of a fermentation medium composed of LB broth supplemented with 10 g/L tryptone, 5 g/L yeast extract, and 10 g/L NaCl. Prior to sterilization, each Erlenmeyer flask received an addition of 8 g/L CaCO_3_. Both the glucose stock solution and the fermentation medium were sterilized independently. The seed culture was then introduced into the fermentation medium, along with the necessary antibiotics, at a concentration of 2%. The cultures were agitated for 3 h at 37 °C and 220 rpm. Subsequently, the shaker’s temperature was adjusted to 30 °C, and 0.5 mM IPTG was introduced to facilitate product synthesis.

### 2.3. Homology Modeling and Molecular Docking

The theoretical models for the native EcHpaB structure were produced utilizing AlphaFold2 [25]. The initial protein structure was manipulated using AutoDockTools version 1.5.6, with the protein’s native charge retained, to produce a pdbqt file suitable for the docking procedure. The ligand *p*-coumaric acid was inserted into the binding pocket of EcHpaB utilizing the AutoDock 4.2.6 software, with the conformation exhibiting the lowest energy within the most prominent cluster being regarded as the most likely natural complex model [26]. Energy optimization was conducted employing the Amber14 force field.

### 2.4. Site-Directed Saturation Mutagenesis

*E. coli* served as the host organism for screening EcHpaB mutants through HPLC analysis. In the creation of EcHpaB mutants, a semi-rational design approach guided by structural information was adopted. In this context, the protein structure was modeled using AlphaFold2 and subsequently docked with *p*-coumaric acid. First, saturation mutagenesis was performed at predicted sites for enhancement in EcHpaB (with the accession number AAR11357.1) by employing whole plasmid PCR and degenerate primers. NNK-containing degenerate primers were utilized for saturation mutagenesis targeting specific mutation sites, with ‘N’ denoting any of the nucleotide bases T, A, C, or G, and ‘K’ referring to T or G. To remove the original template DNA, the PCR amplification products were subjected to *Dpn*I digestion at 37 °C for 1 h, followed by a direct transformation into *E. coli* BL21(DE3) competent cells to generate the variant strains. Successful mutations were subsequently introduced into *E. coli* BL21(DE3) cells for the expression of the EcHpaB protein.

### 2.5. Fermentation of Caffeic Acid in a 5 L Bioreactor

Fermentation of the engineered strain for caffeic acid production was carried out in a 5 L bioreactor (BG-LabSeries 5L, Shanghai Baotech. Engineering Co., Ltd., Shanghai, China). The inoculation proportions of the seed culture were 6% in the fermentor. The composition of the growth medium included 40 g per liter of glucose, 7.5 g/L K_2_HPO_4_·3H_2_O, 1.6 g/L (NH_4_)_2_SO_4_, 1.6 g/L MgSO_4_·7H_2_O, 0.00756 g/L FeSO_4_·7H_2_O, 2 g/L citric acid, 0.02 g/L Na_2_SO_4_, 0.0064 g/L ZnSO_4_, 0.0006 g/L Cu_2_SO_4_·5H_2_O, 0.004 g/L CoCl_2_·6H_2_O, and 100 μg/mL Amp. The medium’s pH was set to 6.8 by titrating with NH_3_·H_2_O throughout the biotransformation procedure. The temperature was maintained at 37 °C with an air flow rate of 2 L per minute. Upon the optical density at 600 nm (OD_600_) reaching approximately 15, the fermenter’s temperature was adjusted to 30 °C. Concurrently, 0.5 mM IPTG and 10 g/L of tyrosine were introduced to the culture to induce enzyme expression and the synthesis of caffeic acid. Samples were collected at defined intervals, and the remaining tyrosine, *p*-coumaric acid, and caffeic acid concentrations were quantified via HPLC analysis. Analytical methods.

Tyrosine, *p*-coumaric acid, and caffeic acid were analyzed and determined using HPLC (Agilent Technologies 1200 series, Hewlett-Packard, Beijing, China). The samples obtained from the shake flask fermentation were diluted to a factor of two with methanol, agitated vigorously, and then centrifuged at 12,000× *g* for a duration of 10 min. Similarly, samples from the 5 L fermenter were diluted 20 times with methanol and subjected to the same centrifugation process. Following centrifugation, the samples were filtered through a 0.22 μm membrane. Analysis of the samples was performed using a reverse-phase C18 column (4.6 × 150 mm, Thermo, Waltham, MA, USA) at a temperature of 40 °C. The mobile phases consisted of buffer A (a mixture of water and 0.1% TFA) and buffer B (a mixture of acetonitrile and 0.1% TFA). The gradient elution program was as follows: from 0 to 10 min, an increase from 10% to 60% buffer B; from 10 to 20 min, an increase from 60% to 80% buffer B; from 20 to 23 min, a decrease from 80% to 10% buffer B; and from 23 to 26 min, maintained at 10% buffer B. The detection wavelength for caffeic acid was fixed at 323 nm, with a flow rate of 1 mL/min [20].

### 2.6. Statistics

The assay values represent the average of three independent experiments, and the error bars represent standard errors. Statistical analysis was carried out by using Student’s *t*-test (one-tailed; two-sample unequal variance; *p* = not significant (ns), * *p* < 0.05).

## 3. Results

### 3.1. Construction of a Heterogeneous Pathway for Caffeic Acid Production in E. coli

**Figure 2:**
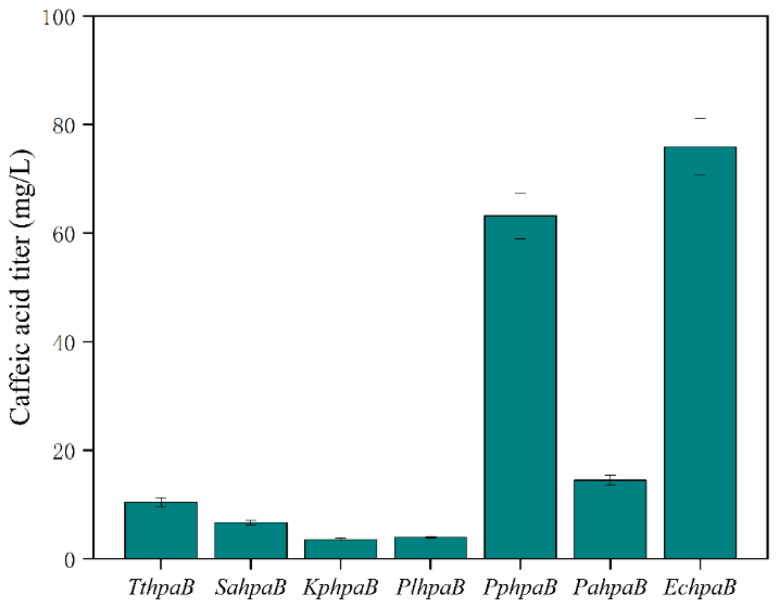
The effect of EchpaC combined with other hpaBs on caffeic acid production.

In this study, a functional heterologous pathway for the production of caffeic acid from tyrosine was established in *E. coli* using a system expressing multiple enzymes. The biosynthetic pathway is depicted in Figure 1. To achieve the biosynthesis of caffeic acid in *E. coli*, a tyrosine ammonia lyase mutant RgTal from Rhodotorula glutinis, HpaB from various origins, and the native HpaC enzyme from *E. coli* BL21(DE3) were chosen for the construction of expression vectors. The proposed heterologous pathway for caffeic acid production comprises two steps. The first step, tyrosine is transformed into p-coumaric acid via the expression of RgTal. The second step was to convert *p*-coumaric acid into caffeic acid mediated by HpaB and HpaC. HpaB has been proven to be the bottleneck enzyme in the production of caffeic acid [16]. In order to study the impact of combining EchpaC with hpaBs from diverse origins on the production of caffeic acid, we employed seven hpaB genes derived from *Thermus thermophiles*, *Sulfobacillus acidophilus*, *Klebsiella pneumoniae*, *Photorhabdus luminescens*, *Pseudomonas putida*, *Pseudomonas aeruginosa*, and *E. coli* in combination with EchpaC. As shown in Figure 2, the strain ZJ02, ZJ03, ZJ04Z, ZJ05, ZJ06, ZJ07 and ZJ08 produced 10.38 mg/L, 6.67 mg/L, 3.54 mg/L, 3.95 mg/L, 63.22 mg/L, 14.46 mg/L and 75.88 mg/L of caffeic acid, respectively. The detected caffeic acid concentrations were notably higher at 75.88 mg/L and 63.22 mg/L when utilizing EchpaB and PphpaB, respectively. However, other combinations yielded significantly lower caffeic acid yields, varying between 3.54 and 14.46 mg/L. These findings conclusively demonstrate the viability of the caffeic acid heterologous pathway in *E. coli*.

### 3.2. Molecular Docking Studies

**Figure 3:**
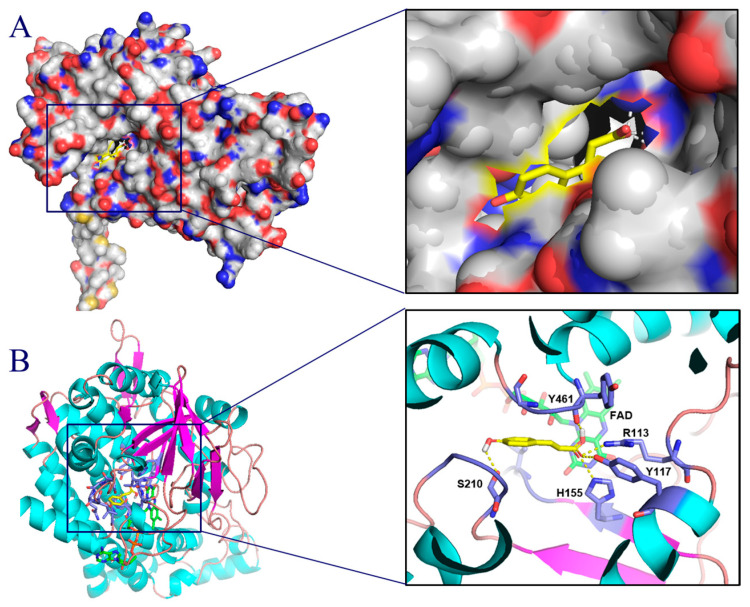
Structural modeling of EchpaB (**A**) and molecular docking (**B**).

To gain a deeper understanding of the molecular interaction between EchpaB and *p*-coumaric acid, a molecular docking investigation was conducted. The results presented in Figure 3 reveal that, following 108 precise simulations, 12 primary binding sites on EchpaB were identified, with the most robust binding site exhibiting a binding energy of −4.95 kcal/mol. In adherence to the principle of minimum energy, the conformation characterized by the least binding energy was chosen as the ultimate outcome of the molecular docking process. This is depicted in Figure 3B. During the molecular docking process, p-coumaric acid predominantly interacted with EchpaB within a hydrophobic cavity, establishing hydrophobic interactions with the adjacent amino acids, under a binding energy of −4.95 kcal/mol.

As shown in Figure 3B, the docking results of EchpaB and *p*-coumaric acid showed that *p*-coumaric acid established five hydrogen bonds with the side chains of R113, Y117, H155, S210 and Y461. Furthermore, the interaction between EchpaB and *p*-coumaric acid is likely due to hydrophobic forces, as evidenced by the formation of robust hydrophobic interactions between *p*-coumaric acid and the residues L93, Y132, Y143, and R145.

### 3.3. Iterative Saturation Mutagenesis of EchpaB to Improve the Production of Caffeic Acid

Despite our successful construction of an artificial biosynthetic pathway for caffeic acid, the introduction of RgTal, EchpaB, and EchpaC into *E. coli* BL21(DE3) (ZJ08) and subsequent cultivation of *E. coli* ZJ08 in a flask resulted in only a minimal accumulation of caffeic acid in the culture. To boost the yield of caffeic acid, our objective is to enhance the catalytic efficiency of the rate-limiting enzyme EchpaB. In pursuit of this goal, we created a mutant library of EchpaB using a semi-rational design approach to augment its catalytic performance. Employing 3D structure-based and molecular docking techniques for enzyme design has been shown to streamline the process of constructing and screening mutation libraries, thereby reducing the overall workload [27,28]. Therefore, sites R113, Y117, H155, S210 and Y461 of EchpaB were selected for subsequent saturation mutagenesis.

**Figure 4:**
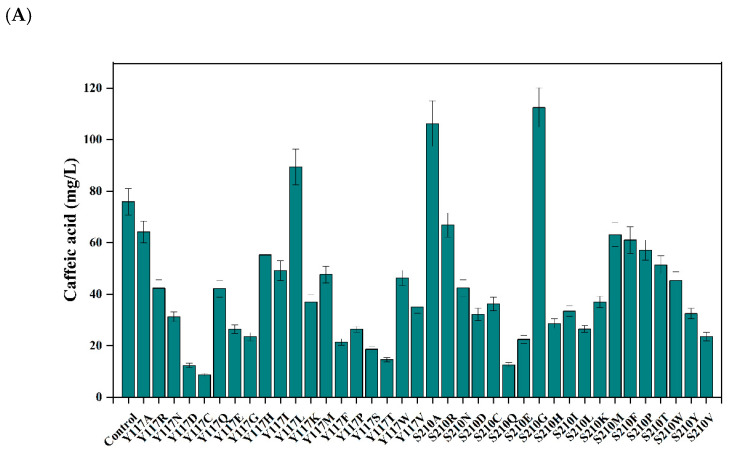

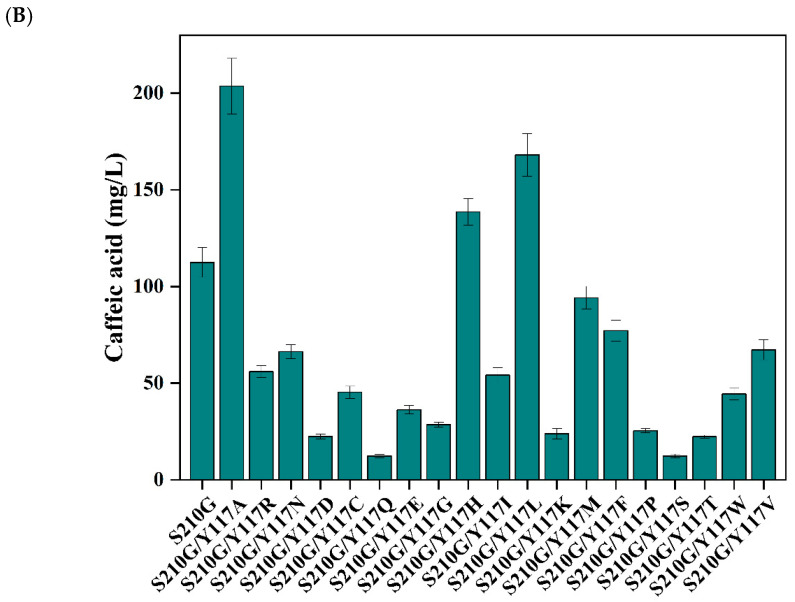
Effects of EchpaB saturation mutations on caffeic acid production. The effects of Y117 and S210 saturation mutations on caffeic acid production (**A**); The effects of S210G-based Y117 mutation on caffeic acid production (**B**).

In the creation of EchpaB mutants, we adopted a semi-rational design approach guided by structural considerations. However, the enzyme activity assays indicated that the saturation mutations at positions R113, H155, and Y461 did not lead to an increase in the enzyme’s specific activity. The fermentation showed that the majority of mutants at positions Y117 and S210 exhibited a decrease in caffeic acid production in the recombinant *E. coli* strains, with three mutants notably influencing caffeic acid yield. The recombinant strains harboring the mutations Y117L, S210A, and S210G demonstrated significantly elevated levels of caffeic acid production compared to the wild type (as shown in Figure 4). Notably, the S210G mutant achieved the highest caffeic acid titer, reaching 112.45 mg/L. Consequently, residues Y117 and S210 were chosen for further iterative saturation mutagenesis studies. These insights enhanced our comprehension of the structure–function relationship of EchpaB and boosted its catalytic efficiency, paving the way for its potential use in industrial applications. Iterative saturation mutagenesis was started at the Y117 site. S210G had the highest activity, yielding 112.45 mg/L of caffeic acid, which corresponds to a 0.48-fold increase relative to the control (Figure 4A). Then, based on S210G, the Y117 site was mutated. Among them, S210G/Y117L, S210G/Y117A, and S210G/Y117H showed higher caffeic acid titers than S210G (Figure 4B), but other mutants showed low caffeic acid titers. The S210G/Y117A variant demonstrated the greatest production level, with the caffeic acid titer reaching 203.64 mg/L, representing a 0.81-fold enhancement when compared to the S210G alone (Figure 4B).

### 3.4. One-Pot Biotransformation of Caffeic Acid Production at the 5 L Fermenter

**Figure 5:**
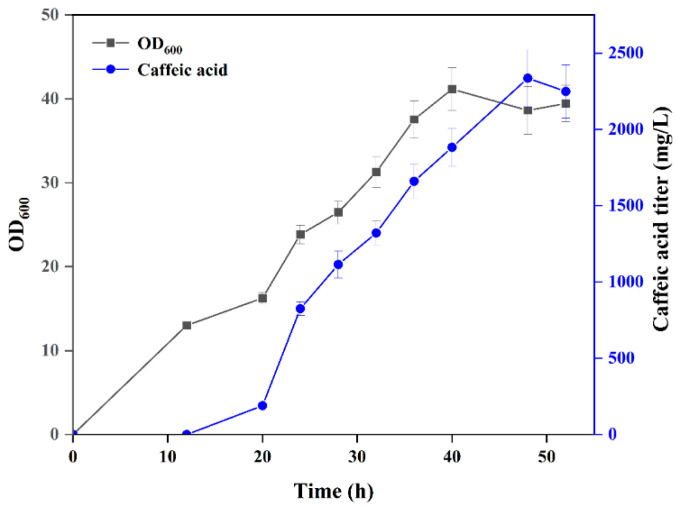
Biosynthesis of caffeic acid by S210G/Y117A in a 5 L fermenter.

To evaluate the capacity for caffeic acid production, the strain S210G/Y117A, which exhibited the most superior performance, was cultivated in a 5 L fermenter (Figure 5). In this study, the biotransformation of caffeic acid was carried out in *E. coli* strain S210G/Y117A, and the findings are presented in Figure 5. The data revealed an increase in biomass, with the OD_600_ value rising from 13 to 23, over the course of 12~24 h. Following 36 h of fermentation, a concentration of 1659.88 mg/L of caffeic acid was accumulated. Extending the fermentation period to 40 h yielded a slightly higher titer of caffeic acid at 1882.16 mg/L. Under the most favorable biotransformation conditions, a caffeic acid concentration of 2335.48 mg/L was achieved in a 5 L fermenter after 48 h.

## 4. Discussion

As previously reported, the concept of facilitating the transformation of *p*-coumaric acid into caffeic acid within *E. coli* has been substantiated as practical [29]. In order to test the presence and functionality of these heterogeneous enzymes in *E. coli*, many different HpaBs have been applied to produce caffeic acid. For example, the enzymes HpaB and HpaC, originating from *Pseudomonas aeruginosa*, were successfully expressed in *E. coli*, leading to the creation of the SyBE_Sc03020005 strain. Following shake flask fermentation, the concentration of caffeic acid achieved a level of 68.2 mg/L [16]. Tian et al. Identified and characterized a novel phenolic acid-responsive transcription factor, CarR, and engineered it into a *p*-coumaric acid biosensor. Finally, the engineered strain CA8 achieved a caffeic acid titer of 9.61 g/L in a 5 L bioreactor [30]. Of course, there are also some studies that use *S. cerevisiae* for heterologous production of caffeic acid. It is indeed possible to produce caffeic acid, but the titer is very low, some are only a dozen mg/L [31], and some are even only a few mg/L [32], which is far lower than the production of caffeic acid by heterologous production of *E. coli*.

Molecular docking analysis revealed that *p*-coumaric acid is capable of binding to the hydrophobic pocket of EchpaB, facilitated by the establishment of hydrogen bonds and hydrophobic interactions with adjacent amino acid residues.

Currently, there is a scarcity of research dedicated to enhancing caffeic acid production via enzyme engineering approaches. In contrast, past studies have typically relied on metabolic engineering techniques to boost caffeic acid yields, encompassing strategies such as heterologous expression of pivotal genes [33,34,35], utilization of co-culture systems [36] and the screening of efficient transporters [20]. Recently, Wang et al. developed a co-culture system to produce caffeic acid using *E. coli* and Candida glycerinogenes as chassis [36]. Finally, 871.9 mg/L of caffeic acid was produced by optimizing the inoculation ratio of co-culture strains [36]. Wang et al. found that polyphenol transporters could promote the production of caffeic acid [20]. The enzyme engineering strategy may involve iterative saturation mutagenesis targeting identified key residues to augment the activity of the bottleneck enzyme. In this study, the S210G/Y117A variant achieved the highest caffeic acid titer. Given that S210 is situated within the substrate pocket, the S210G mutation likely alters the shape of the hydrophobic cavity within this pocket.

Artificial intelligence is becoming a core enabling tool in synthetic biology, accelerating the full chain of intelligence from gene design, enzyme engineering to microbial cell factory construction through deep mining of multi omics data, and promoting the upgrading of biomanufacturing to an efficient and low-carbon customized production paradigm. At the same time, in the food industry, artificial intelligence will further integrate consumer insights with industrial internet of things data, accurately driving the development of personalized nutritional diets, flavor targeted regulation, and intelligent preservation [37], reshaping the technological path and consumer experience of the food industry.

## 5. Conclusions

In conclusion, a biotransformation system that employs Tal, phaB, and phaC to transform tyrosine into caffeic acid within *E. coli* has been constructed. The establishment of this synthetic pathway represents a move towards a more environmentally friendly and health-conscious method of caffeic acid production. This study began with the prediction of the EchpaB protein structure, pinpointing critical mutation sites within EchpaB. Following this, a double mutant, S210G/Y117A, was developed through iterative saturation mutagenesis, resulting in a 1.68-fold enhancement in caffeic acid production. Finally, 2335.48 mg/L of caffeic acid was produced in a 5 L fermenter over 48 h. This study contributes significantly by identifying a superior enzyme component for the single-step fermentation of caffeic acid, and it also offers valuable insights for subsequent metabolic engineering efforts aimed at enhancing caffeic acid yield. The microbial conversion process employs a sustainable substrate and features straightforward culture requirements while minimizing environmental contamination.

## Figures and Tables

**Table 1:** List of strains and plasmids used in this study.

Strains or Plasmids	Relevant Genotype or Description	Source
Strains		
DH5α	Wild type	Invitrogen
*E. coli* BL21(DE3)	Wild type	Invitrogen
ZJ01	BL21(DE3) harboring pTrc99a-RgTal	This Study
ZJ02	BL21(DE3) harboring pTrc99a-RgTal-TthpaB-EchpaC	This Study
ZJ03	BL21(DE3) harboring pTrc99a-RgTal-SahpaB-EchpaC	This Study
ZJ04	BL21(DE3) harboring pTrc99a-RgTal-KphpaB-EchpaC	This Study
ZJ05	BL21(DE3) harboring pTrc99a-RgTal-PlhpaB-EchpaC	This Study
ZJ06	BL21(DE3) harboring pTrc99a-RgTal-PphpaB-EchpaC	This Study
ZJ07	BL21(DE3) harboring pTrc99a-RgTal-PahpaB-EchpaC	This Study
ZJ08	BL21(DE3) harboring pTrc99a-RgTal-EchpaB-EchpaC	This Study
Plasmids		This Study
pTrc99a-RgTal	pTrc99a carries a tyrosine ammonia lyase (with mutants) from *Rhodotorula glutinis*, Amp^R^	
pTrc99a-RgTal-EchpaB-EchpaC	pTrc99a carries a tyrosine ammonia lyase (with mutants) from *Rhodotorula glutinis*, 4-hydroxyphenylacetate 3 monooxygenase HpaB from *E. coli*, and NADPH-flavin oxidoreductase HpaC from *E. coli*, Amp^R^	This Study
pTrc99a-RgTal-SahpaB-EchpaC	pTrc99a carries a tyrosine ammonia lyase (with mutants) from *Rhodotorula glutinis*, 4-hydroxyphenylacetate 3 monooxygenase HpaB from *Sulfobacillus acidophilus*, and NADPH-flavin oxidoreductase HpaC from *E. coli*, Amp^R^	This Study
pTrc99a-RgTal-KphpaB-EchpaC	pTrc99a carries a tyrosine ammonia lyase (with mutants) from *Rhodotorula glutinis*, 4-hydroxyphenylacetate 3 monooxygenase HpaB from *Klebsiella pneumoniae*, and NADPH-flavin oxidoreductase HpaC from *E. coli*, Amp^R^	This Study
pTrc99a-RgTal-PlhpaB-EchpaC	pTrc99a carries a tyrosine ammonia lyase (with mutants) from *Rhodotorula glutinis*, 4-hydroxyphenylacetate 3 monooxygenase HpaB from *Photorhabdus luminescens*, and NADPH-flavin oxidoreductase HpaC from *E. coli*, Amp^R^	This Study
pTrc99a-RgTal-PphpaB-EchpaC	pTrc99a carries a tyrosine ammonia lyase (with mutants) from *Rhodotorula glutinis*, 4-hydroxyphenylacetate 3 monooxygenase HpaB from *Pseudomonas putida*, and NADPH-flavin oxidoreductase HpaC from *E. coli*, Amp^R^	This Study
pTrc99a-RgTal-PahpaB-EchpaC	pTrc99a carries a tyrosine ammonia lyase (with mutants) from *Rhodotorula glutinis*, 4-hydroxyphenylacetate 3 monooxygenase HpaB from *Pseudomonas aeruginosa*, and NADPH-flavin oxidoreductase HpaC from *E. coli*, Amp^R^	This Study
pTrc99a-RgTal-TthpaB-EchpaC	pTrc99a carries a tyrosine ammonia lyase (with mutants) from *Rhodotorula glutinis*, 4-hydroxyphenylacetate 3 monooxygenase HpaB from *Thermus thermophilus*, and NADPH-flavin oxidoreductase HpaC from *E. coli*, Amp^R^	This Study

## Data Availability

Data are contained within the article.
